# Contribution of Topological Domains and Loop Formation to 3D Chromatin Organization

**DOI:** 10.3390/genes6030734

**Published:** 2015-07-27

**Authors:** Vuthy Ea, Marie-Odile Baudement, Annick Lesne, Thierry Forné

**Affiliations:** 1 Institut de Génétique Moléculaire de Montpellier, UMR5535, CNRS, Université de Montpellier, 1919 Route de Mende, 34293 Montpellier cedex 5, France; E-Mails: vuthy.ea@igmm.cnrs.fr (V.E.); mbaudement@igmm.cnrs.fr (M.-O.B.); 2 Laboratoire de Physique de la Matière Condensée, CNRS UMR 7600, UPMC, Sorbonne Universités, 75252 Paris, France

**Keywords:** chromatin dynamics, topological domains, TAD borders, chromatin loops, CTCF, statistical helix

## Abstract

Recent investigations on 3D chromatin folding revealed that the eukaryote genomes are both highly compartmentalized and extremely dynamic. This review presents the most recent advances in topological domains’ organization of the eukaryote genomes and discusses the relationship to chromatin loop formation. CTCF protein appears as a central factor of these two organization levels having either a strong insulating role at TAD borders, or a weaker architectural role in chromatin loop formation. TAD borders directly impact on chromatin dynamics by restricting contacts within specific genomic portions thus confining chromatin loop formation within TADs. We discuss how sub-TAD chromatin dynamics, constrained into a recently described statistical helix conformation, can produce functional interactions by contact stabilization.

## 1. Introduction

In eukaryotic species, the assembly of DNA and histones into chromatin is essential to genome compaction and functions. Observations with optical and electron microscopy led to a classical bipartite description of eukaryotic chromatin. The gene-rich euchromatin is permissive to transcription and positioned at the center of the nucleus while heterochromatin, having a darkish shade, corresponds to a more compact/gene-poor form of chromatin and is generally positioned at the nuclear periphery. One can further distinguish two types of heterochromatin: constitutive heterochromatin is conserved from one cell type to another while facultative heterochromatin is not.

**Figure 1:**
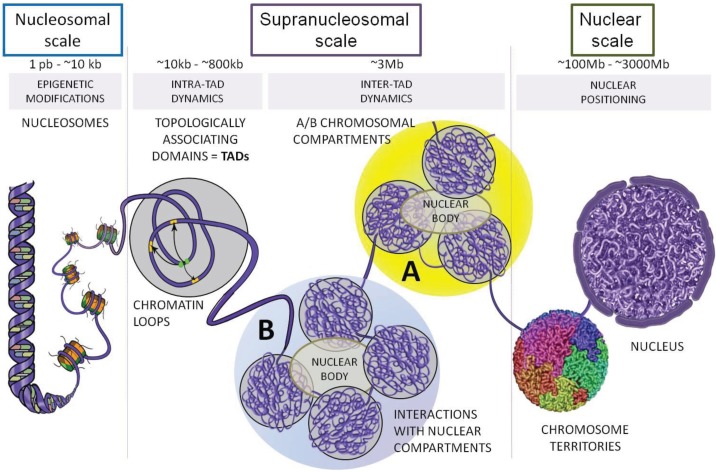
Schematic representation of genome organization in mammals. Between the nucleosomal scale (nucleofilament) and the nuclear scale (chromosome territories), 3D organization of the genome at the supranucleosomal scale has been recently explored thanks to 3C-derived methods (see Figure 2). Beyond the transcriptionally active (**A**) or inactive (**B**) chromosomal compartments, Topologically Associating Domains (TADs) and chromatin loops are two essential determinants of eukaryotic genome organization.

These cytological considerations have been recently renewed by the experimental access to the 3D organization of the genome in the nuclear space [1,2]. This exploration revealed that distinct organization levels exist at the supranucleosomal scale, between the nucleosome scale (nucleofilament) and the nuclear scale (chromosome territories) (Figure 1).

**Figure 2:**
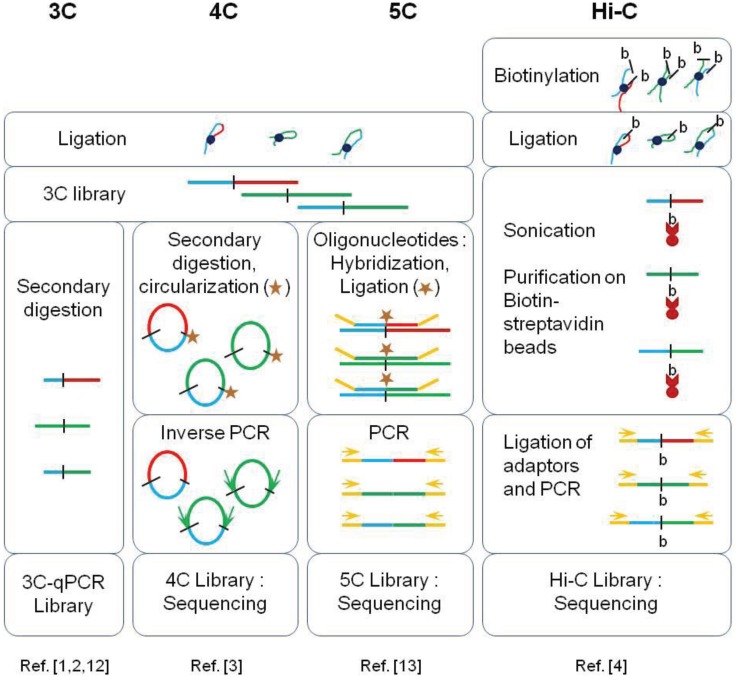
Principles of 3C-derived methods. All the methods derived from the Chromosome Conformation Capture (3C) protocol involve a formaldehyde cross-linking step followed by an enzymatic digestion and a ligation step. Each method uses different approaches to generate genomic libraries: secondary digestion for 3C-qPCR, circularization and inverse PCR for the 4C, “carbon copy” amplification for the 5C and biotinylation and purification on streptavidine beads for Hi-C. Ligation products are quantified by real-time qPCR in the first method or by high-throughput sequencing in the others (adapted from [14]).

This breakthrough was made possible by the development of the Chromosome Conformation Capture (3C) assay and its combination with the Next-Generation Sequencing (NGS) technology allowing the development of the so-called 4C, 5C and Hi-C methods (Figure 2). The advantages and limits of these methods are summarized in Table 1. They essentially differ by the dynamic ranges achieved for quantifying contact frequencies between distant chromatin sites and by the throughput of data generated.

**Table 1:** Advantages and limits of 3C-derived methods.

Method	Genomic Scale Investigated	Advantages	Limits
3C-qPCR	~250 kilobases	Very high dynamic range (highly quantitative), easy data analysis	Very low throughput: limited to few viewpoints in a selected region
4C	Complete genome	Good sensitivity at large separation distances	Genome-wide contact map limited to a unique viewpoint (few viewpoints if multiplex sequencing is used)
5C	Few megabases	Good dynamic range, complete contact map (all possible viewpoints) of a specific locus	The contact map obtained is limited to a selected region
Hi-C	Complete genome	Very high throughput (complete contact map)	Poor dynamic range, complex data processing

The first 4C (Chromosome Conformation Capture on ChIP) experiments, that evaluated for a given region all contacts occurring between this region and the whole genome, showed that chromosomal loci are not only able to establish close contacts in *cis* all along their chromosome, but also in *trans* with other chromosomes [3]. These experiments revealed that such contacts occur in active or inactive “chromatin hubs”. This dichotomy between active and inactive chromatin was then confirmed by Hi-C experiments [4], leading to split the human and mouse genomes into two categories named the A and B compartments (also called “megadomains”, [5]) (Figure 1). After developing a new Hi-C approach named TCC (Tethered Conformation Capture) that considerably reduces the background of inter-chromosomal contacts, Kalhor *et al.* [6] reached similar conclusions. Globally, along the chromosomes, one can distinguish transcriptionally active regions (A-compartments) from inactive regions (B-compartments) (Figure 1). Long-range homologous contacts (A–A or B–B) are largely favored over heterologous contacts (A–B). This organization principle seems to be universal, even if, from one cell type to another, a given chromosomal region can change from a class A compartment to a class B compartment [4].

This dichotomy appears very relevant both structurally and functionally. Indeed, A/B compartments also allow the integration of diverse experimental data [7], such as those issued from transcriptomics or histone modification and protein binding-site maps [8,9]. A-compartments are thus constituted of transcriptionally active gene-rich regions displaying high nuclease sensitivity, while B-compartments are gene-poor, transcriptionally silent and largely insensitive to nucleases [4]. However, this bimodal distribution does not totally reflect the *in vivo* situation since one can observe a continuum between A and B compartments that probably favors compartment switches during cell differentiation processes [10]. Finally, this genomic organization has been proposed to be coordinated through contacts with nuclear compartments or nuclear bodies [11] (Figure 1).

In this review, we present the finer 3D organization of chromatin compartments and discuss its relationships with genome functions and gene regulation. 

## 2. Topologically Associating Domains (TADs)

### 2.1. Identifications of TADs and Their Conservation across Species

The organization of chromatin compartments was further refined in *Drosophila*, in mouse and in human, thanks to 5C and Hi-C experiments [15,16,17,18]. These experiments have shown that chromosomal compartments can in fact be subdivided into domains that display high internal contact frequencies, *i.e.*, contact frequencies of chromosomal regions located within these domains are much higher than across adjacent domains. Because of such preferential contacts, these domains have been named Topologically Associating Domains (TADs) (Figure 1).

The human and mouse genomes are composed of more than 2000 TADs having a median size lower than 1 Mb and covering, altogether, more than 90% of the entire genome (Table 2). TADs have also been evidenced from single-cell Hi-C experiments, indicating that they represent a genuine stable organization principle of mammalian genomes [19].

In the fly *Drosophila melanogaster*, for which genome size is about 30 times smaller than mammalian genomes (~130 Mb *vs.* 3400 Mb in the human), two independent studies have described more than 1100 TADs having a median size of about 60 kb (Table 2): the positions of 42% of TAD borders defined in these two studies coincide at ±4 kb [16,18].

The yeast genomes seem to be devoid of such TAD organization, as shown by Hi-C experiments performed in *Saccharomyces cerevisiae* [20] or in *Schizosaccharomyces pombe* [21] while, in plants, the genome of *Arabidopsis thaliana* appears organized through contacts with pericentromeric regions [22]. However, in these latter two organisms, the existence of domain-like organizations has been suggested [23,24]. Despite these differences, TADs appear as the basic functional units of a chromosome organization that seems widespread at least in the animal kingdom.

TAD organization is also confirmed by cell imaging approaches demonstrating that two loci located in the same TAD are on average closer in 3D space than two loci each located in different TADs [15,25]. More precisely, high-resolution FISH experiments show that two groups of probes tend to superimpose upon one another when they belong to the same TAD, but not when they belong to two different TADs [17].

**Table 2:** Characteristics of TADs identified in Hi-C experiments. Bin size determines the resolution achieved in the experiments. In addition to the references mentioned in this table, other works have been also performed in many other cell types (including neural precursors and astrocytes in the mouse; Hela S3 and HEK293T cells in the human) but without specifying the number and sizes of TADs [26,27,28,29].

Organism	Sample or Cell Line	Number of TADs	Median Size (kb)	Mean Size (kb)	Bin Size (kb)	Reference
*M. musculus*	E14 ESC	2200	880	1093	20–40	[15]
	Cortex	1518	800	1063		
*H. sapiens*	ESC	3127	680	852		
	IMR90	2348	840	1123		
*D. melanogaster*	Embryo	1169	62	100	~10	[18]
	Kc167	1110	61	107	4	[16]

### 2.2. Characteristics of TADs

Remarkably, TAD positions are similar from one cell type to another and they are also highly conserved between mouse and human. TAD stability appears as a powerful means to coordinate chromatin dynamics during cell differentiation. Indeed, in the mouse, cell-specific long-range interactions occurring for separation distances over 20 kb are almost systematically found in the same TAD (less than 4% take place between different TADs) [15]. Furthermore, in *Drosophila*, TADs can be classified according to their epigenetic landscape. At least four classes of chromatin, labeled as “colors”, can be defined: transcriptionally active domains (“red domains”, active domains) are associated to the trimethylation of the lysines 4 and 36 of histone H3 (H3K4me3 and H3K36me3); repressed chromatin includes two kinds of domains, one linked to Polycomb group (PcG) proteins and to trimethylation of the lysine 27 of histone H3 (H3K27me3) (“blue domains”, PcG domains) and the other associated to the HP1 and Su(var)3-9 heterochromatin proteins as well as to dimethylation of the lysine 9 of histone H3 (H3K9me2) (“green domains”, HP1/centromeric domains); finally, the fourth class is not specifically associated to any chromatin mark investigated (“black domains”, null domains) [18]. The euchromatin corresponds to active domains, the constitutive heterochromatin to HP1 domains and the facultative heterochromatin to PcG domains.

### 2.3. TAD Borders

A characteristic feature of TAD borders is that two loci each located on a side of a border establish much less contacts than loci located within the TAD [15,25]. 54% of TAD borders described in mouse Embryonic Stem Cells (ESC) are also found at identical positions in the mouse cortex and 43% of them are conserved in syntenic regions of the human genome. A similar observation was made in two studies performed in *Drosophila*: the first one investigating whole embryos [18] and the second one studying a homogenous cell line (Kc167) [16]. This indicates that borders that demarcate TADs are quite stable structures that possess specific topological characteristics.

However, the mechanisms involved in TAD establishment are not yet fully understood. In mammals, borders are enriched in Transcriptional Start Sites (TSS), more particularly TSSs of housekeeping genes. Accordingly, transcriptional activity in general appears to be high at TAD borders. TAD borders are also enriched in CTCF (CCCTC-binding Factor) insulator protein and Smc1 or Smc3, belonging to the cohesin complex, as well as in active histone marks (H3K4me3 and H3K36me3). Finally, TAD borders are enriched in Med1 and Med12 [15] that belong to the *Mediator* multiprotein complex, which is involved in transcription activation and is known to be important for maintaining cell pluripotency [30]. Similar enrichments are also observed in *Drosophila* where the CP190 insulator protein [31] as well as the *Chromator* factor and DNase hypersensitive sites accumulate at TAD borders. Enrichment in H3K4me3 mark is also found at TAD borders, but in this peculiar case one can observe a 500 bp shift of the enrichment peak toward the active domain [18].

In the mouse, the CTCF protein is found at more than 75% of TAD borders, but only 15% of CTCF sites correspond with TAD borders [15]. Indeed, this zinc-finger protein is highly conserved among mammals where it plays diverse important roles (for reviews see [32,33]). For example, CTCF controls gene expression during the X-inactivation process [34] or the V(D)J recombination in lymphocytes (see [35] for a review). CTCF is also involved at the parentally imprinted *Igf2/H19* locus, leading to distinct 3D organization of the parental alleles [36,37,38].

CTCF depletion affects essentially intra-TAD contacts at separation distances below 100 kb [28,29]. However, TAD borders become much more permissive and inter-TAD contacts are also significantly increased. Therefore, CTCF seems to be involved not only in strengthening intra-TAD interactions but also in preventing long-range contacts between adjacent TADs. These results [29] indicate that some CTCF sites play important roles for maintaining the integrity of TAD borders while so far, the importance of the cohesin complex is less clearly established.

Smc1 and Smc3 are two sub-units of the cohesin complex that also contains the Rad21 and SA1/2 proteins. This complex plays an essential role in maintaining the cohesion between sister chromatids during DNA replication and cell division. CTCF and the cohesin complex map to numerous identical sites on the mammalian genome [30,39,40]. It is therefore not surprising that they are together enriched at TAD borders.

Globally, the insulator proteins identified at TAD borders appear to play a direct structural role in TAD organization [31,41], rather than to block the interactions between functional regulatory elements. Therefore, in that case, the term of “architectural proteins” may be more appropriate [42]. However, the position of a TAD probably also depends on its wider genomic context as shown by the deletion of a 58 kb region removing a TAD border at the X-Inactivation Center in the mouse (∆XTX deletion): this removal perturbs TAD organization but only leads to an incomplete fusion of the adjacent TADs. In this case, other elements, located farther within the domains, contribute to limit inter-TADs contacts [17].

Finally, disruption of TAD borders (e.g., by impairment of binding of associated factors) [28,29] seems to have different architectural and functional effects. This hints at the possible existence of several types of TAD borders.

## 3. Sub-TAD Organization and Contact Domains

Given that TAD borders are highly stable across cell-types, the dynamic regulations of long-range contacts that are required for cell differentiation are expected to take place within TADs. Such contacts, possibly stabilized into cell type-specific interactions, would determine chromatin loops that can contribute to a sub-TAD organization regulated during developmental processes and maintained henceforth. In the mouse, using 5C experiments combined with Next Generation Sequencing (NGS), Phillips-Cremins *et al.* [43] have determined contact frequencies in 6 regions that are 1 to 2 Mb in size around genes important during early development (*Oct4*, *Nanog*, *Nestin*, *Sox2*, *Klf4* and *Olig1-Olig2*). These experiments, which achieved the highest genomic resolution available at that time, allowed identification of around 60 sub-TADs nested within the previously described TADs [43]. Some of them change during the differentiation of ESCs into Neural Progenitor Cells (NPCs) [15]. Furthermore, it was shown that CTCF and cohesin mediate developmentally stable interactions, while CTCF-free contacts often change [43].

Similarly, using 5C experiments, additional sub-TAD structures have been described at the mouse *HoxA* locus that appear to be specific of the developmental stages [44]. Globally, these results strengthen the idea that the genome may be hierarchically organized with a sub-TAD organization in chromatin loops linked to some cell type specificities whereas the TADs would represent stable units maintained throughout cell differentiation and development.

This sub-TAD organization was confirmed in human by high resolution *in situ* Hi-C experiments that identified about 10,000 loops (mostly linked to enhancer/promoter interactions) and defined contact domains having a median size of 185 kb [5]. Interestingly, similar to *Drosophila* TADs, such contact domains can be segregated into at least six classes according to their associated epigenetic landscape. A1 and A2 contact domains are gene dense and linked to active chromatin marks (such as H3K36me3, H3K27ac and H3K4me1), but A2 contact domains have a slightly lower G/C content and are more strongly associated to H3K9me3 than A1 contact domains. B1, B2 and B3 sub-TAD compartments are linked to repressive chromatin. B1 contact domains are linked to facultative heterochromatin, while the B2 compartments mostly include pericentromeric heterochromatin and are strongly associated to Lamina-Associated Domains (LADs) [45,46] and Nucleolus-Associated Domains (NADs) [47]. Finally, the sub-compartment B3 is enriched in LADs but strongly depleted in NADs, while the sub-compartment B4 is restricted to a small 11 Mb portion of chromosome 19 containing both active chromatin (*i.e.*, H3K36me3) and heterochromatin-associated (*i.e.*, H3K9me3) marks [5].

About 65% of such chromatin domains correspond to chromatin loops, the vast majority of which involves a pair of convergent CTCF/RAD21/SMC3 binding sites. Contact domains also tend to be conserved across cell types and between human and mouse [5]. Interestingly, comparative Hi-C in four mammals (mouse/dog/macaque/rabbit) showed that a modular organization of chromosomes is conserved in syntenic regions in relation to the conservation of some CTCF binding sites [48]. Evolutionarily conserved CTCF sites enriched for RAD21 are co-occurring with TAD borders while divergent CTCF binding sites drive loop organization within TADs. Finally, high-resolution 4C-seq showed that conserved CTCF sites display strong directional interactions with neighboring conserved CTCF sites (*i.e.*, evolutionarily conserved orientations of CTCF binding sites) [48].

In conclusion, CTCF appears to have distinct functions in 3D organization of the chromatin: having either a strong insulating role at TAD borders [49,50], or a weaker architectural role in securing chromatin loops [31,51].

## 4. Defining TAD Borders *vs.* Contact Domain Borders and Loop Closures

Globally, the wealth of studies performed in mammals point to a modular model whereby stable TADs are themselves subdivided into multiple sub-TAD structures (chromatin loops) that undergo more dynamic cell-type specific rearrangements. However, our ability to discriminate these two organization levels relies on our ability to define “contact borders”: how can TAD borders be distinguished from a simpler insulating activity mediated by chromatin loops? In Hi-C experiments, this is of course a matter of resolution since chromatin loops, having a relatively small size, can be better evidenced only in the higher-resolution experiments [5]. Moreover, accurate definition of contact borders also depends on our ability to accurately quantify local contact frequencies. It is thus tightly linked to the dynamic range achieved in each experiment, which depends not only on the deepness of sequencing, but also on the complexity of the 3C library. Indeed, while lowering resolution, using a 6-cutter restriction enzyme and binning the data through several tens of kilobases increases the dynamic range of the experiments thus allowing to better evidence TAD borders [15].

**Figure 3:**
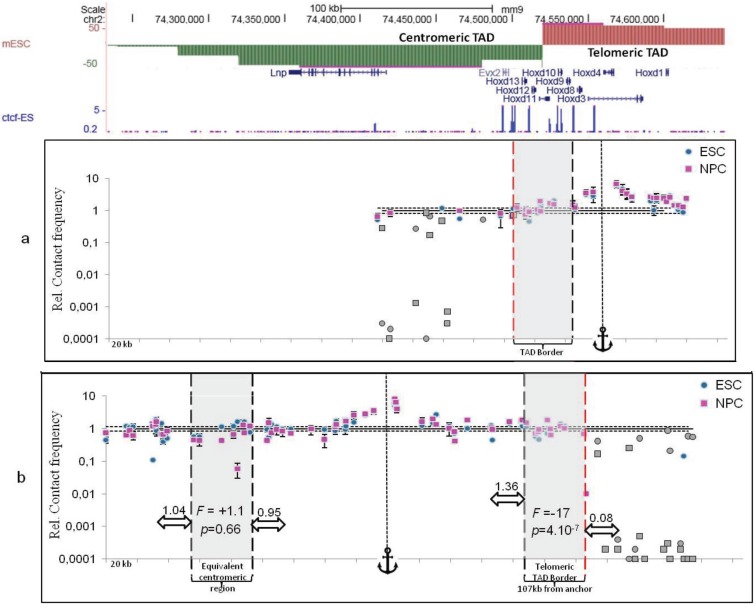
Contact frequencies at the *HoxD* locus in ESC and NPC. Contact frequencies have been determined at the *HoxD* locus (mouse chromosome 2) in mouse ESC (blue circles) and neural progenitor cells (NPC, pink rectangles) using 3C-qPCR as described in [54]. The data have been aligned with the directivity index and the CTCF sites previously described for mouse ESC [15]. The viewpoint used in each 3C experiment is indicated by an anchor. Contact frequencies have been measured from two different viewpoints located in (**a**) the *Hoxd4* gene in the most telomeric TAD (colored in red) or (**b**) the *Lnp* gene in the most centromeric TAD (colored in green). The horizontal lines represent the mean basal contact level (continuous lines) and the associated background noise (dashed lines) [55]. The position of the TAD border is delimited by thick dashed vertical lines (note that this TAD border is delimited by two conserved CTCF binding sites). Error bars correspond to standard error of the mean of five biological replicates each quantified at least in triplicate. Experimental points for which the extremely low contact frequency could not be quantified in all of the five replicates are depicted in grey. In these cases, the points represented in the figure involve an upper bound of the contact frequency corresponding to the detection limits of our qPCR assays: for these points, a Ct of 45 cycles was assigned to replicates that could not be detected by quantitative PCR because of extremely low contact frequencies. These data points were then analyzed according to the same procedure as all the others experimental points as previously described [55]. Mean contact frequencies (values above white horizontal arrows) were calculated over 20 kb on each side of the TAD border (grey rectangle on the right) or on each side of an equivalent region located on the centromeric side of the same viewpoint (grey rectangle on the left). The fold change (ratio) *F* between these mean contact frequencies is indicated together with the *p*-value of their difference (Student *t-*test).

**Figure 4:**
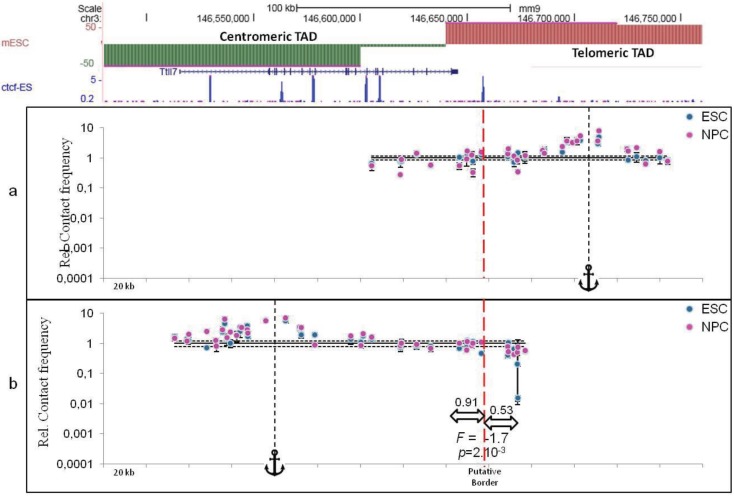
Contact frequencies at the *Ttll7* locus in ESC and NPC. Following the conventions described in Figure 3, contact frequencies have been determined at the *Ttll7* gene locus (mouse chromosome 3) in mouse ESC (blue circles) and neural progenitor cells (NPC, pink circles) from (**a**) an intergenic viewpoint located in the most telomeric TAD (colored in red) or (**b**) from an intragenic (*Ttll7* gene) viewpoint in the most centromeric TAD (colored in green). Error bars correspond to standard error of the mean of five biological replicates each quantified at least in triplicate. The most telomeric CTCF site appears as an interesting element delineating a putative border (dashed red vertical line). The fold change (*F*) between the mean contact frequencies on each side of this putative border and the *p*-value of their difference were determined as explained in the legend of Figure 3.

Finally, the best methods to distinguish TAD borders from chromatin loops are those having the best dynamic range. In that respect, the more classical quantitative 3C approaches [12] remain the best methods to characterize not only local chromatin loops, as largely exemplified in the literature for a vast number of loci (see for example [52]), but also TAD borders. Indeed, performing quantitative 3C experiments through TAD borders identified by Hi-C experiments can greatly help to assess whether such borders are more or less permissive to long-range *cis* inter-TAD contacts. To illustrate the relevance of quantitative 3C approaches, we present experimental results obtained in mouse embryonic Stem Cells (ESC) and Neural Precursor Cells (NPC) at two loci: the *HoxD* gene locus (Figure 3) and the *Ttll7* gene locus (Figure 4). The *HoxD* gene cluster has been described as precisely located at a TAD border [15] and higher resolution experiments have shown that a switch of enhancers between adjacent TADs underlies the collinearity of *HoxD* gene expression observed in mouse limbs [53]. The border located at the *Ttll7* gene locus was identified in previous Hi-C experiments [15] but was not further characterized. For each locus, contact frequencies were determined from two viewpoints (anchors in the figures) located on the telomeric or centromeric sides of the borders (see Figure 3 and Figure 4, a and b panels, respectively). At the *HoxD* gene locus, contact frequencies fall dramatically 61.6 kb upstream the telomeric viewpoint (red vertical dashed line in Figure 3a) and 127 kb downstream the centromeric viewpoint (red vertical dashed line in Figure 3b). This allows mapping the border within a gene-rich 38 kb large region delimited by two CTCF sites. In contrast, at the *Ttll7* gene locus, no drastic decrease could be evidenced, neither from the telomeric (Figure 4a) nor from the centromeric (Figure 4b) viewpoints. Therefore, while a large typical TAD border could be easily evidenced at the *HoxD* gene locus, no strong border could be defined at the *Ttll7* locus. However, a unique CTCF site located at the expected position may display the property of a weak border (red vertical dashed line in Figure 4b).

As our experiments are highly quantitative, we could take advantage of that feature to better characterize these two putative types of border. To this aim, we calculated the mean contact frequencies 20 kb upstream to the borders and we compared them with the mean contact frequencies observed 20 kb downstream. The strong TAD border observed at the *HoxD* gene locus, located 107 kb from the viewpoint on the telomeric side, is associated with a very strong decrease in contact frequencies: the mean contact frequency observed downstream to this border is 17 fold lower than that observed upstream to the border (Figure 3b, grey rectangle on the right). We also checked the significance of the difference between these means, as given by its *p*-value (*p* = 4.10^−7^, Student *t-*test). Importantly, an equivalent region, located 107 kb on the centromeric side of the same viewpoint and displaying no evidence for any border, displays no significant change (fold change of 1.1 and difference with *p*-value of *p* = 0.66) (Figure 3b, grey rectangle on the left). These results demonstrate that the decrease observed on the telomeric side of this viewpoint corresponds to a specific feature linked to the presence of a strong border preventing long-range contacts between adjacent TADs.

In contrast, mean contact frequencies across the putative border identified at the *Ttll7* locus (CTCF site) display a fold change of only 1.7 (Figure 4b). While the difference between these mean contact frequencies is significant (*p* = 2.10^−3^, Student *t-*test), their fold change is at least 10 times weaker than that observed for the TAD border of the *HoxD* locus (Figure 3b). This result confirms the presence of a weak border at this locus that may be explained, for instance, by the presence of a chromatin loop.

Globally, these results are also fully consistent with our previous observation [54] indicating that, in gene rich regions as those investigated here, contact frequencies are modulated with a period of 100 kb and that, consequently, contact frequencies of sites located around 100 kb are stable (Figure 3b, grey rectangle on the left) except, of course, if a strong TAD border (Figure 3b, grey rectangle on the right) or a weak border (Figure 4b) interferes with this underlying chromatin dynamics.

**Figure 5:**
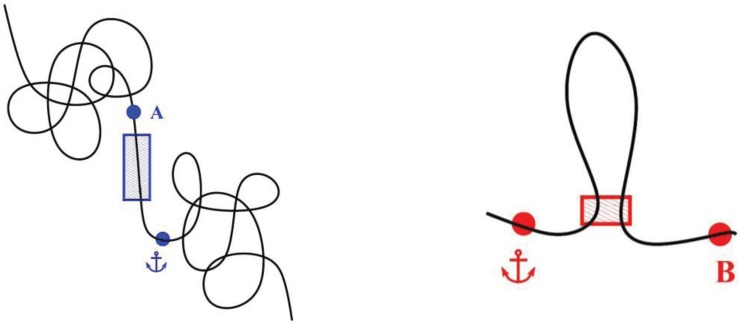
Two different kinds of borders: TAD borders and loop closures. The figure depicts a border separating two adjacent TADs along the genome, sketched as a blue box (**Left**), and a chromatin loop and its closure by stabilizing factors, sketched as a red box (**Right**). When performing a quantitative 3C experiment with the viewpoint represented by the anchor, TAD border plays a strong insulating role by preventing contacts between the viewpoint (anchor) and point A, as observed in Figure 3, thus demarcating two adjacent domains. In contrast, the formation of a chromatin loop, while locally increasing the contact frequency between the two stretches of DNA in the stem region (red rectangle), weakly lowers (or does not affect) the contact frequency between the anchor and point B. This weak-border signature may correspond to that seen in Figure 4.

Interestingly, as shown above, the *HoxD* cluster thus appears as a highly structured locus, even in cells where it is repressed. Therefore, similar to sub-TAD chromatin loops, TAD borders can sometimes appear as specialized 3D structures extending over several tens of kilobases. However, TAD borders are stable structures and no or very low contacts can be detected through them (Figure 5).

**Figure 6:**
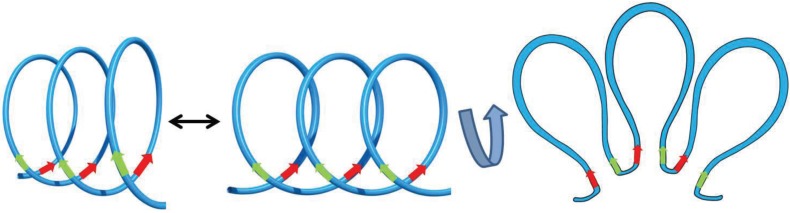
From statistical helix to 2D representation of chromatin loops. As recently shown [36,54], spontaneous conformational fluctuations of the chromatin fiber are constrained at supranucleosomal scale, which produces a helical statistical shape. Specific factors stabilize statistical contacts (*i.e.*, random collisions), turning them into stable interactions and creating 3D topological loops of chromatin [59]. 2D projection of such 3D rosette-like structures (as sketched by the curved arrow) leads to the current plane representation of chromatin loops [5,58]. Green and red arrows depict putative CTCF sites involved in chromatin loop formation and represent the directionality of these sites [5,48].

In contrast, sub-TAD 3D organization is characterized by its highly dynamic nature. Chromatin loops, mediated by stable locus-specific interactions, occur in that fluctuating context [56] (Figure 5). At this scale, conformational fluctuations of the chromatin fiber are primarily constrained by its own stiffness, which favors or restricts the formation of long-range contacts, as argued in Kleckner [57] in the context of meiosis. In interphase, with no anchoring of the chromatin loops onto a scaffold, the array of long-range contacts when observed in a large population of cells, as in 3C experiments, tends to fold statistically the chromatin into a helical shape [54] (Figure 6, left part). This 3D organization is fully compatible with rosette-like structures (Figure 6, middle part) and is naturally prone to form chromatin loops provided that their closure may be achieved by stabilizing factors (Figure 5). However, for the sake of simplicity, such rosette-like structures are usually represented as a 2D projection taking the form of the chromatin loops classically represented in most models [5,58] (Figure 6 right part).

## 5. Conclusions

As the resolution, the dynamic range and the panel of methods available for studying chromatin dynamics increase, the organization principles of the eukaryote genomes become more and more clear. Our current view on eukaryote genomes points to a compartmentalized multi-scale organization that controls chromatin dynamics to restrict or favor intra- or inter-chromosomal contacts [11]. While TADs now appear as an essential unit of genome organization, exploring chromatin dynamics at the sub-TAD/chromatin-loop level is now essential to unravel the tight relationships that should exist between the functions and the fluctuating 3D organization of the eukaryote genomes.
